# Regional Anesthesia as an Emergency Department Consulting Service: A Quality Improvement Pilot Study

**DOI:** 10.7759/cureus.95836

**Published:** 2025-10-31

**Authors:** Rodney A Gabriel, John J Finneran, Michael A Orcutt, Dale Bongbong, Jessica Oswald

**Affiliations:** 1 Anesthesiology, University of California San Diego, San Diego, USA; 2 Emergency Medicine, University of California San Diego School of Medicine, San Diego, USA; 3 Pain Management, University of California San Diego School of Medicine, San Diego, USA

**Keywords:** acute pain in emergency department, emergency medical service, regional anesthesia, regional nerve blocks, safe regional anesthesia

## Abstract

Purpose: Despite evidence that regional anesthesia may facilitate earlier discharge and lower outpatient opioid utilization, a multitude of systemic and cultural barriers reduce the utilization of regional anesthesia in the emergency setting. An accessible regional anesthesia team was created to assess Emergency Department (ED) utilization of nerve blocks and their impact on patient pain improvement.

Materials and methods: ED providers in a non-trauma hospital had a regional anesthesia team available as a consulting service for five months. Patients were enrolled based on inclusion criteria and at the discretion of the ED attending. Enrolled patients underwent peripheral nerve blocks with one of two anesthesiologists who were fellowship trained in Regional Anesthesiology. Patients were then contacted on post-procedure day 1 and were interviewed regarding their satisfaction with the process and their ultimate post-ED disposition.

Results: Participation was limited due to multiple systemic and cultural factors. The majority of patients enrolled participated in the follow-up survey and rated their pain relief as 'Very Good' or 'Excellent' (n=9/14). Nearly all patients (n = 11/14) responded ‘Strongly Agree’ to whether they would use the same type of pain relief method in the future, and most (n=11/14) rated their overall experience with their pain management service as ‘Excellent’ or ‘Very Good’.

Conclusion: This pilot study showed that implementation of a regional anesthesia consulting service in the ED was feasible and that the majority of patients were satisfied with the quality of care and the timeframe in which they were provided regional anesthesia for acute pain.

## Introduction

In the setting of the ongoing opioid epidemic in the United States, multi-modal pain management has become the standard of care both for its superior pain relief over standard opioid regimens [1] and for decreasing opioid utilization in the outpatient setting. Regional anesthesia procedures are an additional non-opioid modality that may have the potential to facilitate discharge and reduce the need for opioid prescriptions in the Emergency Department (ED); however, regional anesthesia and acute pain teams have historically limited their scope of practice primarily to patients undergoing surgical procedures or admitted patients. In many healthcare systems, workflow mechanisms do not exist to connect regional anesthesia providers to patients in the ED [2]. Thus, patients in the ED are more likely to receive alternative analgesics (i.e., opioids), which have been demonstrated to be inferior in decreasing pain intensity compared to regional anesthetic procedures [3]. The Society of Academic Emergency Medicine published a 2021 article outlining a curriculum for training ED physicians to perform regional anesthetics and encouraging collaboration between ED departments and regional anesthesiologists in the interest of attaining optimal standards of care when performing regional anesthesia in the ED [2]. 

Our institution recently undertook a pilot program to assess the feasibility of providing regional anesthesia to both non-surgical and surgical patients presenting to the ED with acute pain. This pilot program consisted of a collaborative workflow (with the use of an electronic medical record secure chat system) in communication between the ED physicians and the regional anesthesia team. Here we present the initial findings from this patient quality improvement initiative.

## Materials and methods

**Figure 1:**
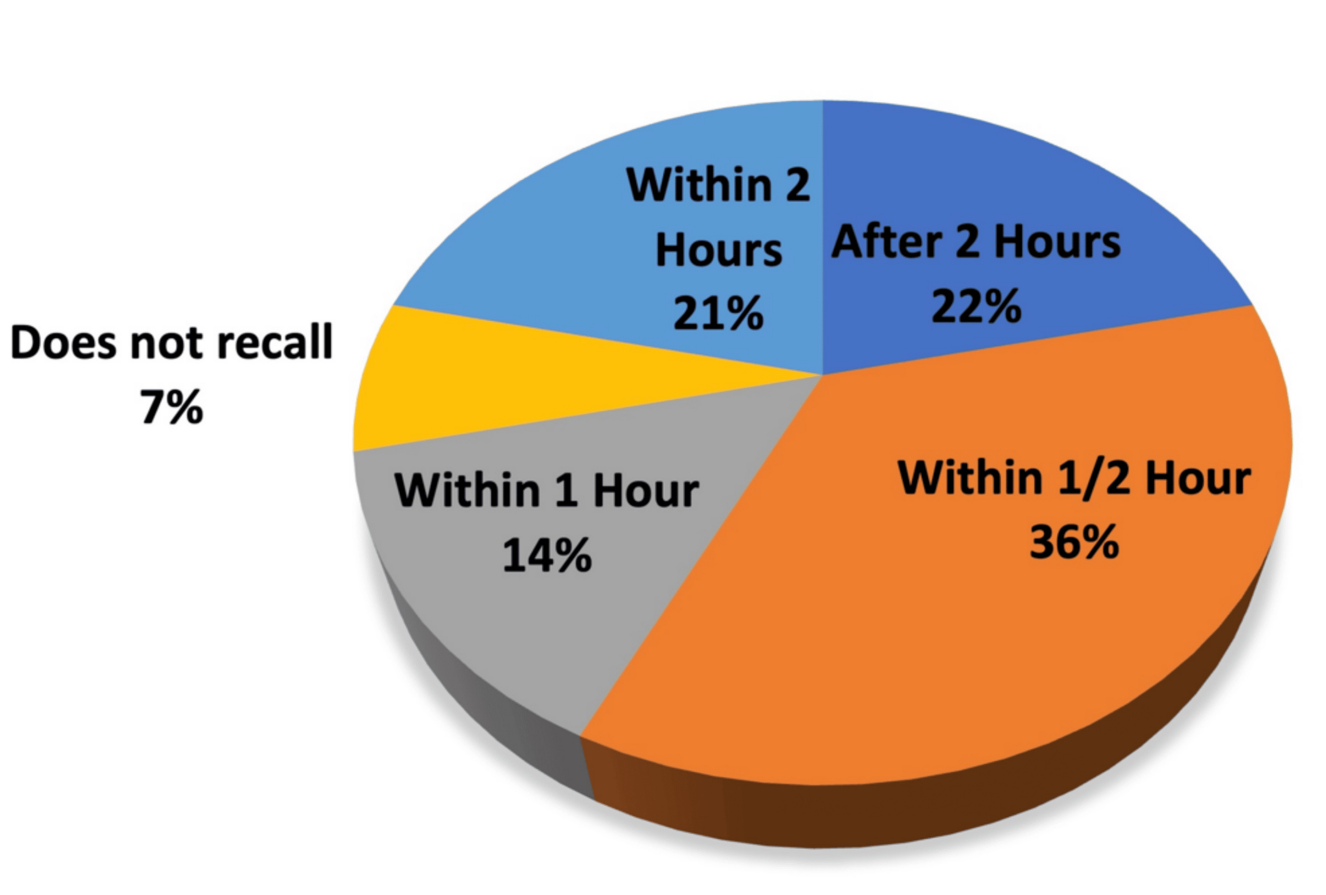
Time to Pain Team Arrival Follow-up survey results of Survey Question 1) When you were in the emergency department, the pain team responded

The protocol was submitted to our institutional review board and was deemed IRB-exempt as a quality improvement project. ED providers at the participating hospital were informed of the pilot study and were instructed to contact the Regional Anesthesia team via secure chat in the local EMR if they wanted the patient to be evaluated for an intervention. Patient eligibility criteria included adult patients (>18 years at the time of presentation) with acute pain localized to a single area of the body. Patients were evaluated with a history and physical exam and if clinically indicated, patients were then offered a nerve block by one of two anesthesiologists, both of whom are fellowship-trained in Regional Anesthesiology and Acute Pain with more than five years of experience. If patients had guests in the department, they were given the option to remain present if desired by the patient. Type of nerve block was determined by the anesthesiologist based on the location of the patient’s pain and the expected duration of the pain. The blocks performed included local anesthetic-based blocks (erector spinae plane; femoral, obturator; occipital, paravertebral, sciatic, and fascia iliaca) with both single injection and continuous techniques, as well as cryoneurolysis of the intercostal nerves for chest wall pain. Patients received telephone follow-up on the day following the block, which is part of our institution’s normal standard of care practice following a nerve block (Table 1). These interviews were conducted by a male Pain fellow. The interview consisted of questions about patients’ experience pertaining to the nerve block, level of pain relief, and patient experience with the pain management team (Figure 1). No audio or video recording of these interviews was performed. Patients’ discharge location from the ED (e.g., home, operating room, or admitted for pain control) was also recorded (Figure 1). 

**Table 1:** Follow-up Patient Survey Questions and answer choices from the follow-up patient survey.

Question	Answer Choices
When you were in the emergency department today, the pain team responded ______.	Within ½ hour, within one hour, within two hours, after two hours
What was the quality of pain relief after the pain team intervention?	Excellent, Very Good, Good, Fair, Poor
How would you rate the attentiveness and sensitivity of the pain team staff?	Excellent, Very Good, Good, Fair, Poor
How was your overall experience with your pain management service?	Excellent, Very Good, Good, Fair, Poor
I would use the same type of pain relief method in the future:	Strongly Disagree, Somewhat Disagree, Neither Agree nor Disagree, Somewhat Agree, Strongly Agree
I would recommend the same type of pain relief method to family/ friends:	Strongly Disagree, Somewhat Disagree, Neither Agree nor Disagree, Somewhat Agree, Strongly Agree
The pain team was courteous and professional during your interaction:	Strongly Disagree, Somewhat Disagree, Neither Agree nor Disagree, Somewhat Agree, Strongly Agree

## Results

**Figure 2:**
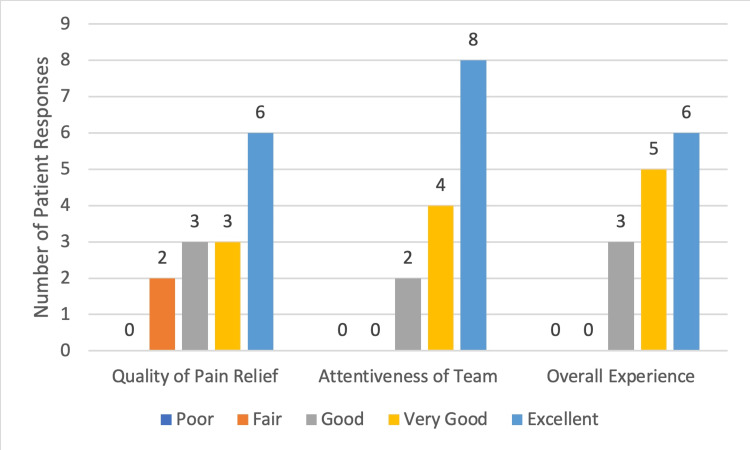
Subjective Patient Experience with Pain Team Results of Survey Questions 2-4: 2) What was the quality of pain relief after the pain team intervention, 3) How would you rate the attentiveness and sensitivity of the pain staff, 4) How was your overall experience with your pain management staff?

**Figure 3:**
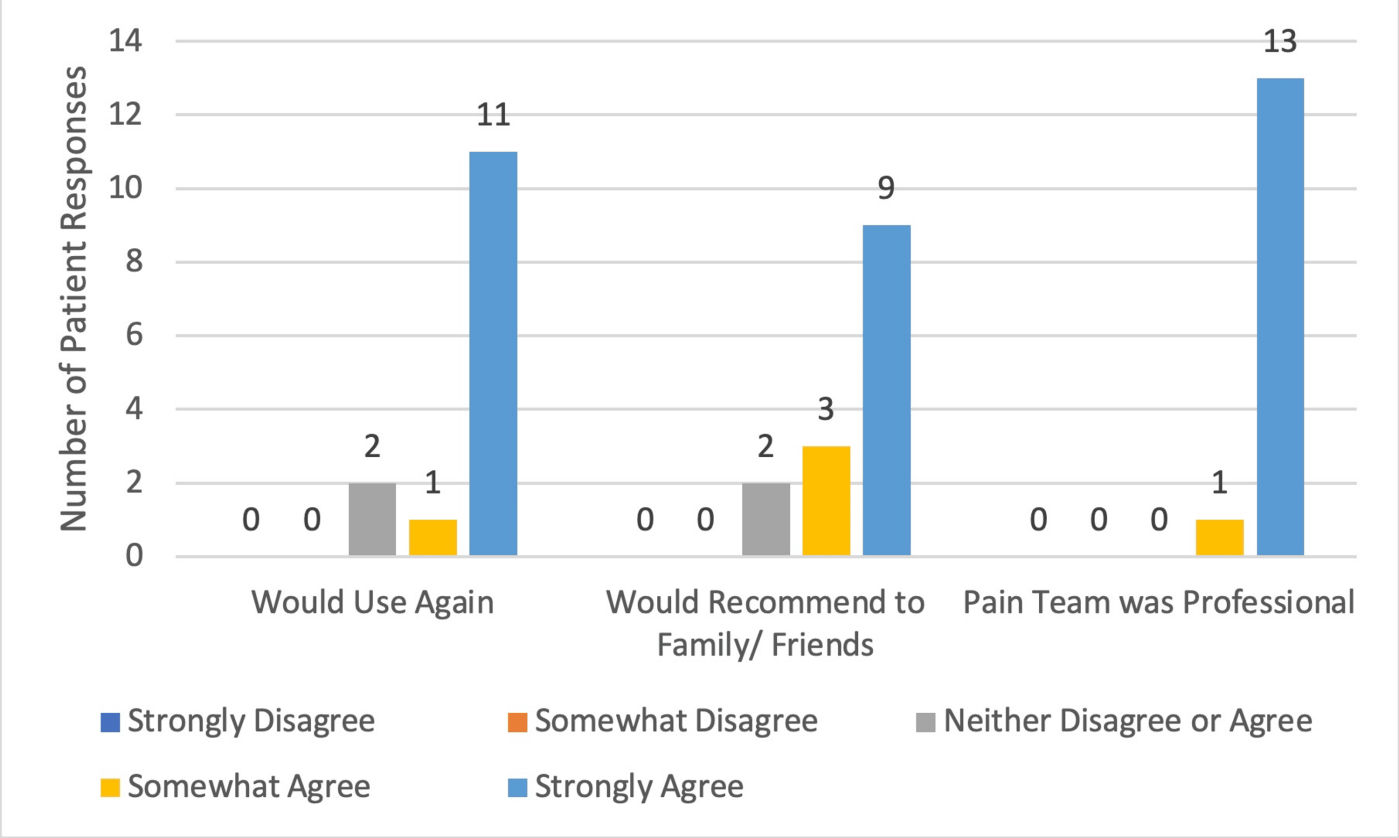
Patient Evaluation of Pain Team Results of Survey Question 5-7: 5) I would use the same type of pain relief method in the future, 6) I would recommend the same type of pain relief to family/friends, 7) The pain team was courteous and professional during your interaction.

**Figure 4:**
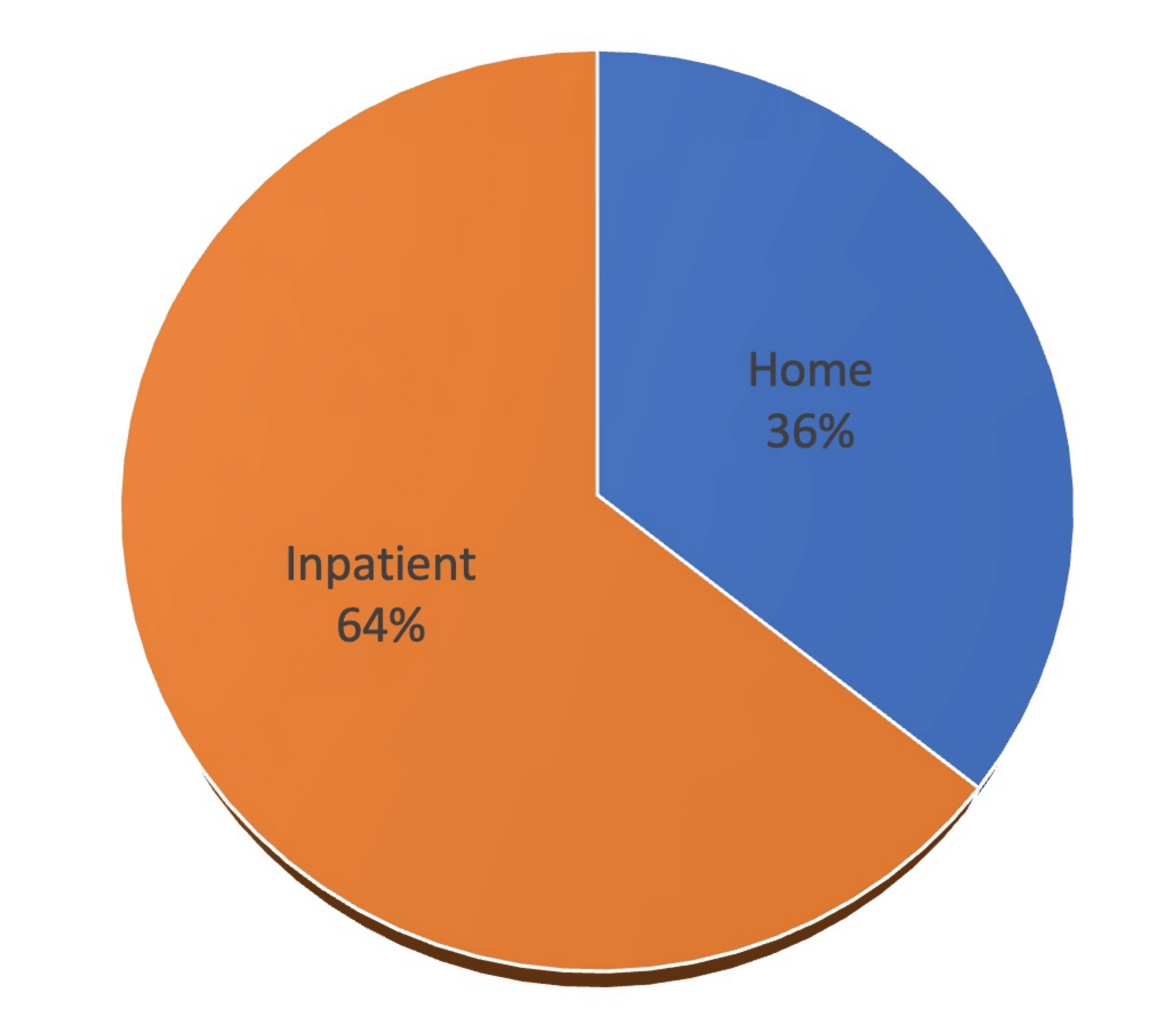
Patient Disposition Discharge location from the ED (home or inpatient admission for pain management or surgical management).

A total of 22 patients received regional nerve blocks between October 13, 2022 and March 21, 2023. Presenting pathology included occipital neuralgia, femoral neck fracture, femoral shaft fracture, rib fracture, humerus fracture, and pelvic fracture. Fourteen patients answered the follow-up patient survey, six patients did not answer after three phone call attempts, one patient was in hospice and was unable to participate in the survey, and one patient declined to answer. Content analysis performed on the aggregate data revealed the following trends. The most common response to the question of how quickly the pain team responded (Question 1) (Figure 1) was within one half hour (n = 5/14), and two more patients answered within one hour. Most patients rated the quality of pain relief after the nerve block (Figure 2) (Question 2) as ‘Excellent’ (n = 6/14) or ‘Very Good’ (n = 3/14) (total n = 9/14 or 64.3%) and no patients rated it as ‘Poor’. Most patients rated their overall experience with their pain management service (Figure 2) (Question 4) as ‘Excellent’ (n = 6/14) or ‘Very Good’ (n = 5/14) (total n = 11/14 or 78.6%). Nearly all patients (n = 11/14 or 78.6%) responded ‘Strongly Agree’ to whether they would use the same type of pain relief method in the future, and no patients disagreed (Question 5). Most patients responded ‘Strongly Agree’ (n = 9/14) or ‘Somewhat Agree’ (n = 3/14) (total n = 12/14 or 85.7%) to whether they would recommend the same type of pain relief method to their family/friends, and none disagreed (Figure 3) (Question 6). Nine patients (64.3%) were discharged home and the remaining four patients (35.7%) were admitted to the hospital for pain control or surgical management (Figure 4).

## Discussion

This pilot quality improvement program demonstrated that regional anesthetics can be performed in a timely manner for patients presenting with acute pain to the ED. The majority of patients reported positive ratings to questions about pain relief and were discharged home, supporting that regional anesthetics have the potential to provide a safe and effective alternative to opioid analgesia. A growing amount of literature suggests that regional anesthesia offers superior outcomes regarding pain improvement and adverse effects in the ED [4-6]. Consideration of this treatment modality for acute pain in both non-surgical and surgical patients is imperative in the setting of the current opioid epidemic. More work in the form of randomized clinical trials, including comparison with standard opioid treatment, would appear to be indicated for further assessment of the feasibility, safety, and effectiveness of regional anesthetics in the ED.

As a pilot study, this project primarily sought to assess the feasibility and utilization of a dedicated regional anesthesia consulting service in the ED as well as the patient experience with such a service. Limitations of utilization included the limited daytime availability of the performing anesthesiologists as well as ED provider awareness of the availability of services. Although ED providers were not formally surveyed as part of the study, multiple providers reported that they often considered blocks for patients outside of the window of availability of the regional services but felt that their workload and lack of formal training prevented them from performing the blocks themselves. Patient enrollment (n=14) didn’t occur at a volume that would warrant or sustain a dedicated emergency medicine regional anesthesia team. Despite low enrollment, patients overwhelmingly reported a positive and timely experience when receiving regional blocks in the ED. The majority of the regional blocks performed as part of this study are within the scope of practice for ED physicians with an expanding body of evidence demonstrating that they are both safe and effective for ED providers to perform [7-9]. As a future study, we hope to perform a randomized controlled trial in which patients would undergo ultrasound-guided peripheral nerve blocks performed by ED physicians who completed training in the appropriate block techniques, although this also would have its own cultural and institutional barriers. Our institution is currently surveying ED providers regarding practice barriers to performing regional anesthesia and is actively engaging in research regarding the effectiveness of self-guided, hands-on training for ED providers. 

## Conclusions

This pilot study showed that implementation of a regional anesthesia consulting service in the ED was feasible and that the majority of patients were satisfied with the quality of care and the timeframe in which they were provided regional anesthesia for acute pain. 
